# Composition of Guayule (*Parthenium argentatum* Gray) resin

**DOI:** 10.1038/s41598-023-29524-w

**Published:** 2023-02-28

**Authors:** Amandine Rousset, Christian Ginies, Olivier Chevallier, Mariano Martinez-Vazquez, Ali Amor, Michel Dorget, Farid Chemat, Sandrine Perino

**Affiliations:** 1 GuaTecs, 28 Rue Xavier Bichat, 72000 Le Mans, France; 2 Avignon University, INRAE, UMR408, GREEN Extraction Team, 84000 Avignon, France; 3 Avignon University, INRAE, UMR408, MicroNut Team, 84000 Avignon, France; 4 Avignon University, DARI, Plateforme 3A, 84000 Avignon, France; 5 Instituto de Química, Universidad Nacional Autónoma de México, 04510 CDMX, México

**Keywords:** Plant sciences, Chemistry

## Abstract

Guayule (*Parthenium argentatum* Gray) is a semi-arid shrub, native from the Chihuahan desert. This plant produces polyisoprene and resin. Polyisoprene is the main focal point of many researches, from structure to properties. Today, some processes are used to extract polyisoprene under its dry form, using solvent extraction, to produce rubber (used in truck or airplane tires) or as an emulsion, to make latex products by dipping (used in medical gloves, condoms, etc.). This article focuses on guayule resin which has some interesting applications in adhesives, coatings, pharmaceuticals, etc. In order to better know the resin composition and to be able to perform comparisons between varieties or seasons, liquid and gas chromatographic analysis methods have been described, for the groups of molecules composing the resin (polyphenols, guayulins, free fatty acids, di and triacylglycerols, argentatins, alkanes, alkanals, sugars, organic acids). Unlike other articles, this study aims to analyze all components of the same resin; the average composition of a guayule resin is given.

## Introduction

Since the pre-Columbian times, guayule has always generated interest: it was common knowledge that this plant produces an elastomer. The main focus has always been on polyisoprene, accounting up to 10% dw (dry weight) of the branches. It can be extracted under two forms: dry polyisoprene to make rubber or as an emulsion to obtain latex. Natural rubber is today a critical raw material because it remains irreplaceable for many applications, such as airplanes tires. Furthermore, guayule latex has the particularity of being non allergenic^1^. Therefore, guayule may seem to be a potential candidate for different polyisoprene applications. In order to be able to sell this rubber at a competitive price, co-product must be valorized. And it turns out that guayule also produces resin, a mix of molecules that can find a lot of applications in adhesives, coatings, perfumes, pharmaceuticals, etc^2^.

**Figure 1:**
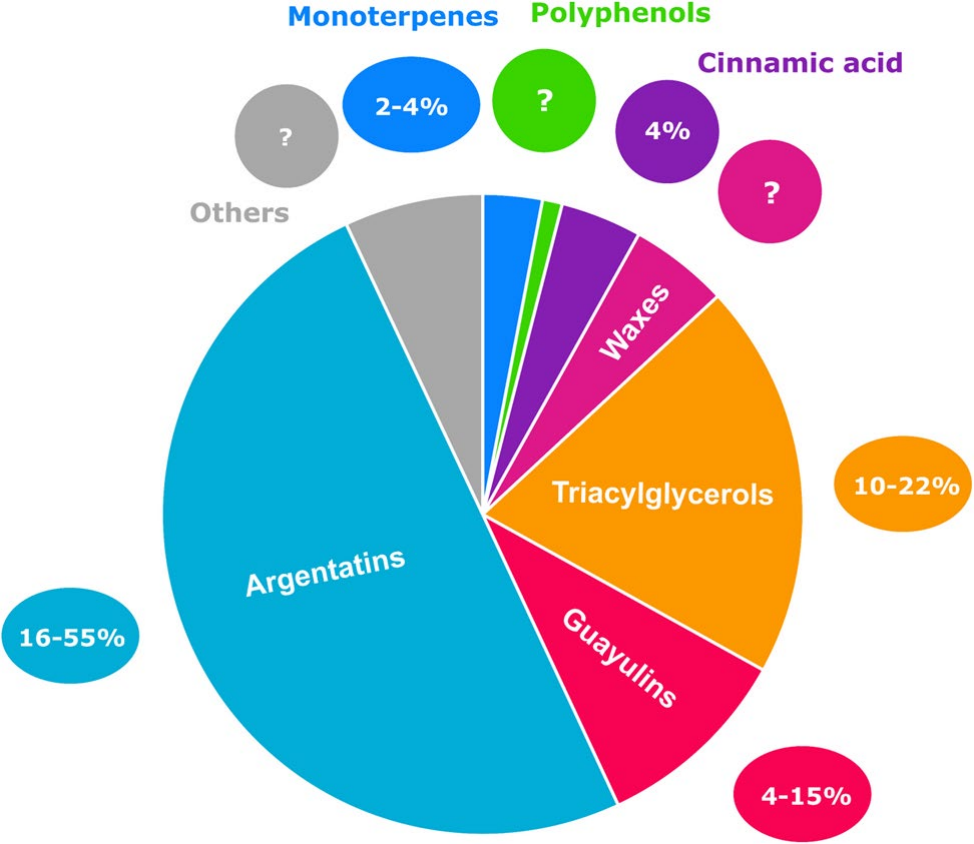
Compounds identified and quantified in Guayule resin (composite view of previous studies).

Most constituents of guayule resin have already been described: polyphenols, guayulins, triglycerides, argentatines, waxes, sugars, organic acids, phytosterols and monoterpenes^3–8^. Polyphenols were found in guayule leaves^3,9^ and detailed lists including more than 30 molecules have been published^10,11^. No quantification of resin polyphenols has been done yet. Guayulins (A, B, C, D) have been well described, and some studies show quantity variations among various cultivars and seasons^4,12,13^ or in the distinct parts of the plant^14^. Two methods are used to quantify: using the corresponding chromophores or with guayulin standards. As for neutral lipids, Schloman was the first to find that the predominant form was triglycerides (with gel permeation chromatography (GPC) using trilinolein as standard)^15,16^ and most authors prefer to give fatty acid composition after derivatization^17^. Argentatins were studied with a lot of attention, due to their particular forms: infrared spectroscopy (IR), nuclear magnetic resonance (NMR)^18–21^, and mass spectrometry (MS) fragments were given^22^. Quantification have been made using high-performance liquid chromatography with ultraviolet detection (HPLC–UV) after reacting with dinitrophenylhydrazine (DNPH) with ter-butylcyclohexanone as a standard^15^. Guayule waxes have not been studied a lot^23^ but Marwah indicated the general composition of waxes extracted from leaves in 1994: 3% of alkanes (C19–C36) and 92% of esters^7^. Sugars were analyzed only in the woody byproduct components obtained after polyisoprene extraction, named bagasse. The goal was to evaluate the fermentable sugars, and the approach was based on the acid hydrolysis process^5^. Last but not least, monoterpenes were found in the essential oil obtained by hydrodistillation from leaves^8,24^, from resin^25^ and a detailed list of monoterpenes in the resin was given by Dehghanizadeh^26^ using gas chromatography with mass spectrometry (GC–MS) and FT-ICR MS (Fourier-transform ion cyclotron resonance with mass spectrometry) Fig. 1 presents a composite view from previous references cited.

The aim of this article is to introduce improved techniques to analyze and quantify the families of molecules composing the resin, in order to be able to make a seasonal and variety composition tracking. Polyphenols, guayulins, argentatins, free fatty acids, di and triacylglycerols were analyzed with liquid chromatography. Sugars, alkanes, alkanals, and organic acids were analyzed with gas chromatography. This study aims to qualify and quantify all the molecule families contained in a resin.

## Materials and methods

### Plant material

Four-year-old guayule plants, lines CL1 (french variety from the original USDA 11,591) and CL3 (USDA N 593) are hand-harvested (5 cm above the ground) and hand-defoliated from a field near Montpellier, France. They are transported at room temperature during few hours, hand-cut to obtain pieces of few centimeters and dried at 50 °C in a laboratory convection oven (Binder oven) to constant weight^27^. Plants are then cut using first a blade crusher (Blixer 2, Robot coupe) and then a coffee grinder (Quilive, Auchan) to obtain pieces smaller than 2 mm. They are stored at room temperature before ASE resin extraction (few days).

### Chemicals and reagents

All solvents were HLPC (High Performance Liquid Chromatography) quality grade from Sigma Aldrich. Standards were also purchased at Sigma Aldrich except argentatins, which were extracted and kindly donated by Professor Martinez-Vazquez, UNAM, Mexico.

### Resin extraction

Resin (defined by acetone extract) is extracted from ground guayule shrub samples using an Accelerated Speed Extractor (ASE) (Buchi Speed Extractor E-914).

**Figure 2:**
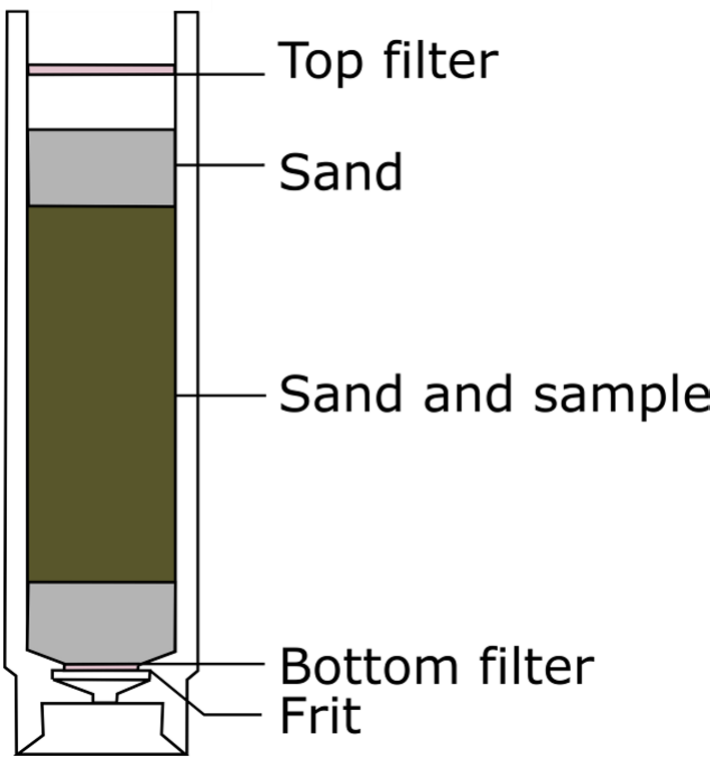
Filling scheme of an ASE cell.

40 mL stainless steel extraction cells with metal frits are filled with three layers: 2 g inert sand (Fat free quartz sand 0.3–0.9 mm (Buchi)) a mix of 5 g of a dry sample and 25 g of sand and 2 g of sand (Fig. 2). Bottom glass fiber filters (Buchi) are inserted into stainless steel extraction cells prior to loading samples into the cells and top cellulose filters are added on the top after.

After preheating at 40 °C, three 20 min acetone (HPLC quality) extraction cycles are applied with a 100% flush each time. Pressure is at 100 bar (with N_2_) and temperature at 40 °C. Acetone extracts are collected in 120 mL vials and then transferred into round-bottom flasks in order to evaporate the acetone, first with Rotavap (BUCHI) and then dried at 50 °C in a laboratory convection oven (Binder) to constant weight. Once weighed, the dried acetone extract is diluted with acetone to obtain a 10 mg/mL solution and stored in the freezer (− 20 °C) until analysis^28^. The dry extract is call “ASE resin”.

### Chromatographic methods

*Polyphenols and guayulins* were analyzed by Ultra-Performance Liquid Chromatography (UPLC) using an ACQUITY UPLC® system (Waters Corp., Milford, MA, USA) linked simultaneously to both a Diode Array Detector 190–800 nm (DAD, Waters, Milford, MA, USA) and a Bruker Daltonics HCT Ultra Ion Trap MS equipped with an electrospray ion source (UPLC DAD/ESI-MSn). Analysis was carried out with a Waters C18 HSS T3 (100 mm × 2.1 mm × 1.7 µm; Waters corp., Milford, MA, USA) column at 40 °C and 0.4 mL/min. The initial mobile phase is composed by 97% solvent A (0.1% formic acid in water) and 3% solvent B (acetonitrile). The percentage of solvent B was then increased to reach 10% at 3 min, 30% at 20 min, 100% at 22 min. Then a linear gradient follows to go back to 3% at 24 min. The ion trap was operated in the Ultra scan mode from m/z 100 to 1000 and spectra MS2 were recorded to identify the compounds. Negative ionization was used with following condition: dry temperature 365 °C, dry gas flow 9 L/min, nebulizer pressure 50 psi and capillary voltage − 2 kV. Quantification was made with UV at 320 nm for the caffeoyls derivatives, 250 nm and 275 nm for guayulins. The results were expressed in equivalent of 5-O-caffeoyl quinic for all the mono caffeoyl quinic acids, 4,5-dicaffeoyl quinic for all the di caffeoylquinic acids^29^. Guayulins were detected in positive mode. Guayulin A and C were quantified by injection of cinnamic acid standard and guayulins B and D, by p-anisic acid standard.

*Free fatty acids, Di and Triacylglycerols*. The same system UPLC-DAD-ESI-MSn was used to identify and quantify the neutral lipids in the extract with the same column. The temperature of the column was 40 °C and the total flow rate 0.4 mL/min. The initial mobile phase consisted of 100% solvent A (acetonitrile:water 80:20 10 mM ammonium formate) and 0% solvent B (isopropanol), which was run during 6 min. The percentage of solvent B was increased to reach 40% at 15 min, 90% at 17 min. The column was then equilibrated with the initial mobile phase composition prior to the next run. Identification was made by negative mode for all the fatty acids (M–H)^−^ and positive mode for the acylglycerols, by detecting the adduct ion [M + NH_4_]^+^. Quantification was made with trilinolein equivalent for the di and triacylglycerols and with all the fatty acids standards.

*Argentatins* were determined using a UPLC-MS system Waters with a C18 CSH Waters (100 m × 2.1 mm × 1.7 µm) column. The temperature of the column was 45 °C and the total flow rate 0.4 mL/min. The initial mobile phase consisted of 95% solvent A (acetonitrile:water 80:20 and 0.1% formic acid) and 5% solvent B (acetonitrile: isopropanol 50:50 and 0.1% formic acid), which was run during 1.5 min. The percentage of solvent B was increased to reach 99% at 16.5 min, then hold up to 18.75 min and decreased to go back to the initial condition at 19 min. Argentatins A standard was used to quantify all argentatins. Data were acquired in positive ionisation mode in high resolution mode from 50 to 1200 m/z with a scan time of 0.08 s/scan using MSe (low energy 4 eV and high energy ramp from 25 to 50 eV). Source temperature was set up at 120 °C with a capillary voltage of 0.8 kV and a cone voltage of 40 V with a gas flow of 50 L/h. Desolvation temperature was 450 °C with a gas flow of 650 L/h. A solution of 1 ng/mL of Leu-Enk was infused at 10 µL/min during each injection in order to correct for accurate mass acquisition. Argentatin A was used to quantify Argentatin A, Incanilin, Argentatin C, Isoargentatin C and one unknown compound and Argentatin B was used to quantify all Argentatin B and Isoargentatins B isomers.

*Alkanals and alkanes* were determined using a GC–MS system (Shimadzu QP2010) with a ZB-5MS Phenomenex (30 m × 0.25 mm × 0.25 µm). Extracts in acetone were diluted with dichloromethane. A 2 µl sample was injected in split mode (ratio 10). The injection temperature was maintained at 300 °C, and Helium was the carrier gas at a constant flow rate of 1.3 mL.min^−1^. The column temperature program consisted initial temperature 60 °C, increases at 20 °C/min to 200 °C, then 5 °C/min to 280 °C, then 3 °C/min to 320 °C, followed by an isothermal hold at 320 °C for 5 min. Mass spectra were recorded in Electronic Ionisation at 70 eV. The scan range was set from 40 to 600 m/z at 0.3 scan.s^−1^. Alkanes were quantified by injection of standard mixture C10–C40 (Merck). When the alkane did not exist in the standard mixture, the closest alkane was used for the quantification. Docosanal was used to quantify all the alkanals.

*Sugars, glycerol and organic acids* were determined using a GC–MS system (Shimadzu QP2010, Kyoto, Japan) with a ZB-5MS Phenomenex (30 m × 0.25 mm × 0.25 µm). 50 µL of 10 mg/mL ASE resin in acetone solution were dried under nitrogen. 50 µL of 20 mg/mL methoxyamine in pyridine were added and heated at 80 °C during 30 min. Then of 80 µL N,O-Bis(trimethylsilyl)trifluoroacetamide (BSTFA) was added and the mix was heated at 80 °C during 30 min.

A 2 µl sample was injected with split mode (ratio 10). The injection temperature was maintained at 300 °C and Helium was the carrier gas at a constant flow rate of 1.3 mL.min^−1^. The column temperature program consisted of injection at 60 °C, temperature increases of 20 °C/min to 200 °C, then 5 °C/min to 280 °C, then 3 °C/min to 320 °C, followed by an isothermal hold at 320 °C for 5 min. Mass spectra were recorded in Electronic Ionisation at 70 eV. The scan range was set from 40 to 600 m/z at 0.3 scan.s^−1^. Compounds are identified by comparison with the mass spectral library NIST 2.3 (2017) and by injecting standards.

## Results and discussion

20 samples of ASE resins from four-year-old plants of two different varieties: CL1 (corresponding to USDA 11591) and CL3 (corresponding to USDA N 593) and from the same field in Lansargues (south of France) were analyzed. The plant samples were collected and extracted from February to December. No trends were observed that could differentiate the two varieties and the results given are an average and biological range between shrubs (same age, two varieties).

### Polyphenols and guayulins

An analytical method to quantify polyphenols and guayulins (sesquiterpene esters) in the same run has been developed. In relation to polyphenolic compounds, six compounds that belong to the family of chlorogenic acids were found: 3-O-caffeoylquinic acid, 4-O-caffeoylquinic acid, 5-O-caffeoylquinic acid, 3,4-di-O-caffeoylquinic acid, 3,5-di-O-caffeoylquinic acid, 4,5-di-O-caffeoylquinic acid. They are quite common in nature and characterized by specific fragmentation patterns in negative mode, given by Clifford^29^.

Three compounds with m/z 353 [M − H]^−^ and UV maximum absorbance at 325 nm were identified, characteristic of caffeoylquinic isomers. The three isomers can be differentiated by their MS2 fragment abondance: 3-O-caffeoylquinic acid (353, 191, 179, 135), 4-O-caffeoylquinic acid (353, 191, 179, 173, 135) and 5-O-caffeoylquinic acid (353, 191, 179, 161). UV confirms the presence of caffeoyl derivative by their characteristic absorbance: 3-O-caffeoylquinic acid (243/325), 4-O-caffeoylquinic acid (219/327), and 5-O-caffeoylquinic acid (219/(237)/325).

For the double radical derivatives, three isomers can be differentiated by their MS2 fragment abondance: 3,4-O-dicaffeoylquinic acid (353, 335, 191, 179, 173), 3,5-O-dicaffeoylquinic acid (353, 191, 179) and 4,5-O-dicaffeoylquinic acid (353, 179, 173). The UV signal gives patterns close to the monoderivatives ones: 3,4-O-dicaffeoylquinic acid (219/(240)/321), 3,5-O-dicaffeoylquinic acid (219/(243)/328) and 4,5-O-dicaffeoylquinic acid (213/(256)/331).

**Figure 3:**
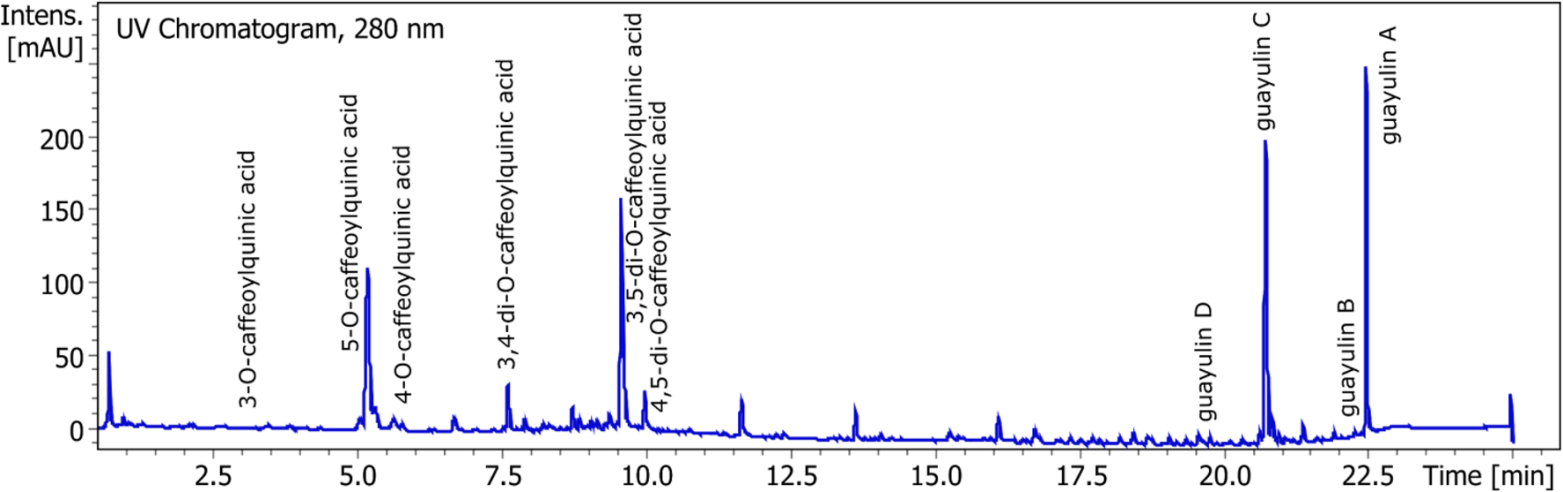
UPLC chromatograph of polyphenols and guayulins.

UV and MS spectra allow to identify guayulins. Maxima of absorbency and order of elution were given by Sidhu, Rozalén and Teetor^4,12,14^: guayulins A and C: 277 nm and guayulins B and D: 256 nm. Guayulins A and C are composed of a cinnamic acid chromophore and guayulins B and D of p-anisic chromophore. Thanks to these previous informations, all guayulins were detected by analyzing the UV signal at 280 nm. Comparison between our spectra with the ones given by Rozalén gave the following results: for Guayulin A, main peaks were found at 351 [M + H]^+^, 203, 149, 147, 131 and for Guayulin C: 367 [M + H]^+^, 349 [M − H_2_0]^+^, 219, 201, 159, 147, 131, respectively. (Fig. 3 and Table 1) Guayulins A and C consist of the chromophore of cinnamic acid and anisic acid respectively, bound to the patheniol form. Due to the absence of the hydroxyl group from the spatulenol form, their signal in positive mass detection is weak^4^. Guayulins B and C contain the presence of this hydroxyl group, but concentrations in samples are low.

**Table 1:** Identification of polyphenols and guayulins in ASE resin.

Name	Formula	λ_max_	[M − H]^−^, [M + H]^+^	Fragments	Standard equivalent
3-O-caffeoyl quinic acid	C_16_H_18_O_9_	325	353 [M** − **H]^−^	191 [M − C_9_H_6_O_3_]^−^, 179, 135	Chlorogenic acid
5-O-caffeoylquinic (chlorogenic) acid	C_16_H_18_O_9_	325	353 [M** − **H]^−^	191 [M − C_9_H_6_O_3_]^−^, 179, 161	Chlorogenic acid
4-O-caffeoyl quinic acid	C_16_H_18_O_9_	327	353 [M** − **H]^−^	191 [M − C_9_H_6_O_3_]^−^, 179, 173, 135	Chlorogenic acid
3,4-di-O-caffeoylquinic acid	C_25_H_24_O_12_	321	515 [M** − **H]^−^	353, 335, 191, 179, 173	4,5-di-O-caffeoylquinic acid
3,5-di-O-caffeoylquinic acid	C_25_H_24_O_12_	328	515 [M** − **H]^−^	1031 [2 M − H], 353, 191, 179	4,5-di-O-caffeoylquinic acid
4,5-di-O-caffeoylquinic acid	C_25_H_24_O_12_	321	515 [M − H]^−^	353, 179, 173	4,5-di-O-caffeoylquinic acid
Guayulin A	C_24_H_30_O_2_	277	351 [M + H]^+^	203, 149, 147, 131	Cinnamic acid
Guayulin B	C_23_H_30_O_3_	256	355 [M + H]^+^	203,149	Methoxybenzoic (p-anisic) acid
Guayulin C	C_24_H_30_O_3_	277	367 [M + H]^+^	349 [M − H_2_0]^+^, 219, 201, 159, 147, 131	Cinnamic acid
Guayulin D	C_23_H_30_O_4_	256	371 [M + H]^+^	353 [M − H_2_O]^+^, 219, 201, 159, 147	Methoxybenzoic (p-anisic) acid

**Figure 4:**
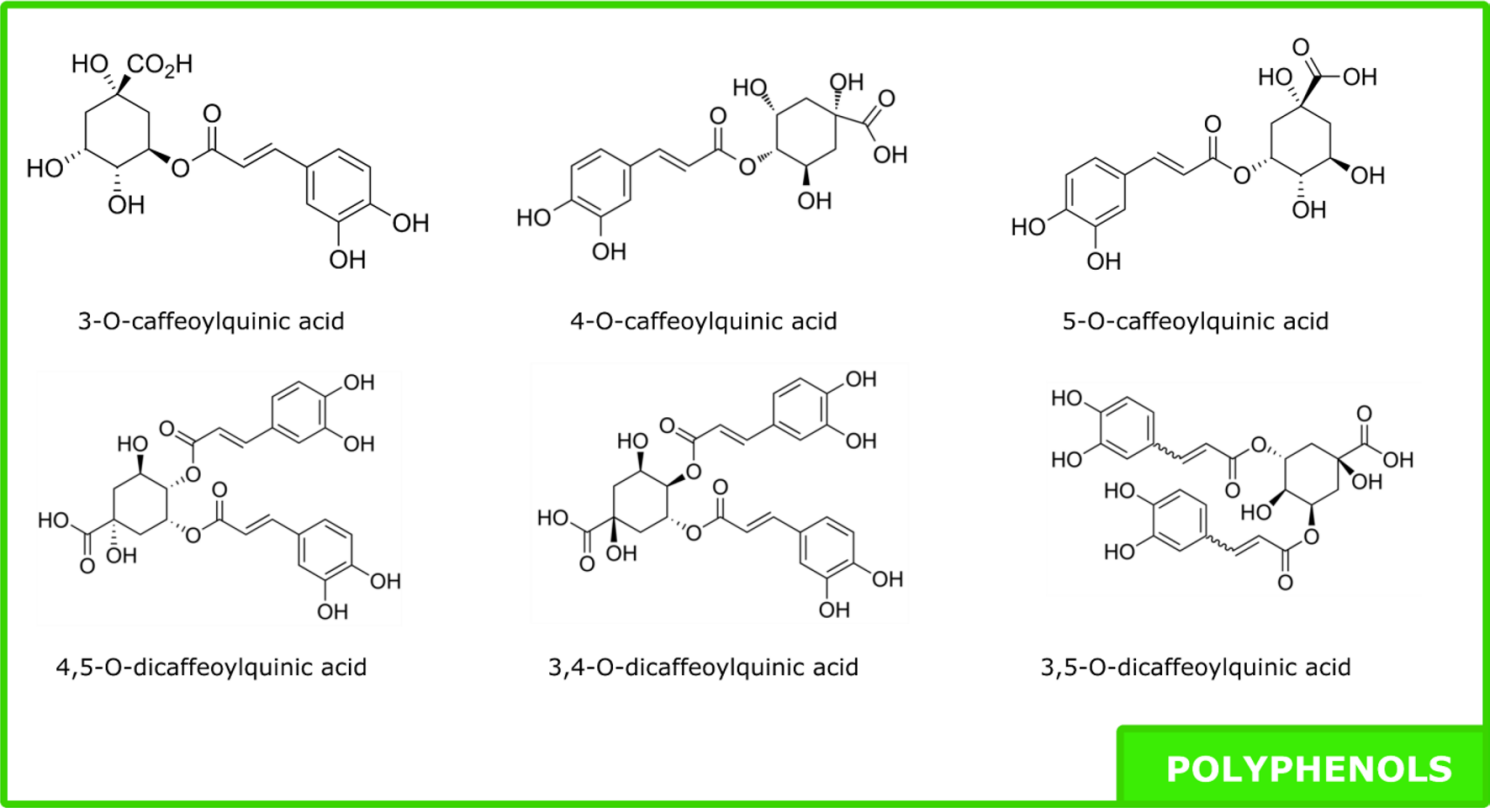
Molecules of polyphenol family found in ASE resin.

**Figure 5:**
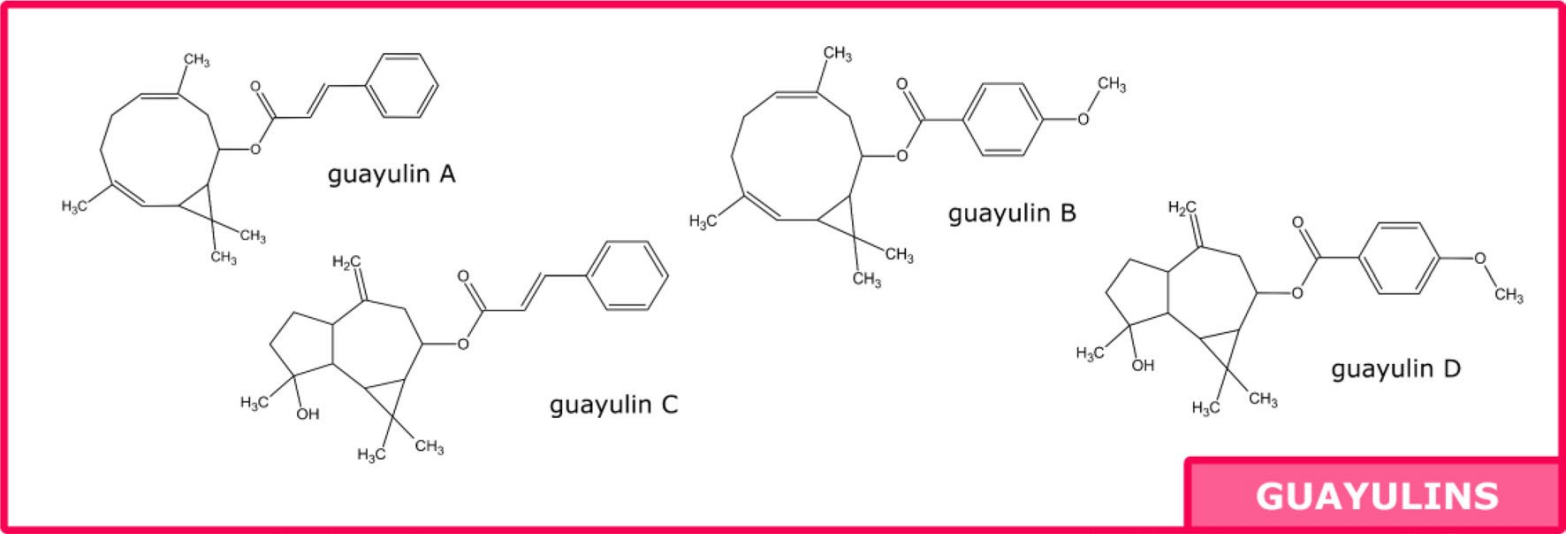
Molecules of guayulin family found in ASE resin.

Guayulins (A, B, C, D) content was between 4.1% and 12.1%; 8.6 ± 2.4% in average and it was mainly guayulins A and C (> 70% for every sample) (Figs. 4 and 5).

A study of 13 cultivars of 4-year-old plants in India^12^ found that Guayulin A content accounts for 0.7 to 5.9% of the ASE resin coming from the entire plant (and not only from stems) and Guayulin B content, between 0.1% and 1.8%. Guayulins are present at higher proportions in guayule stems^14^, so these results are consistent with those obtained here. As Rozalén describes^4^ in an article, “guayulin similars” were also found in some samples but in too little amount to be quantified. This same team worked on neighbor joining trees clustering the content of guayulins A, B, C and D and total guayulins. They placed CL-1 (corresponding to CL1 here) and 593 (corresponding to CL3 here) on related branches. Model groups including CL1 and CL3 varieties, of two-year-old plants contain guayulin contents between 8.18 and 9.3% of the ASE resin. The results obtained are comparable with those given by previous authors even if quantification is different. They both used guayulins extracted in the laboratory as analytical standards.

As for polyphenols, less than 1% in total (0.01 to 0.9%) were found. To the authors’ knowledge, the quantification of polyphenols in the ASE resin had not yet been carried out.

### Free fatty acids, di and triacylglycerols

**Figure 6:**
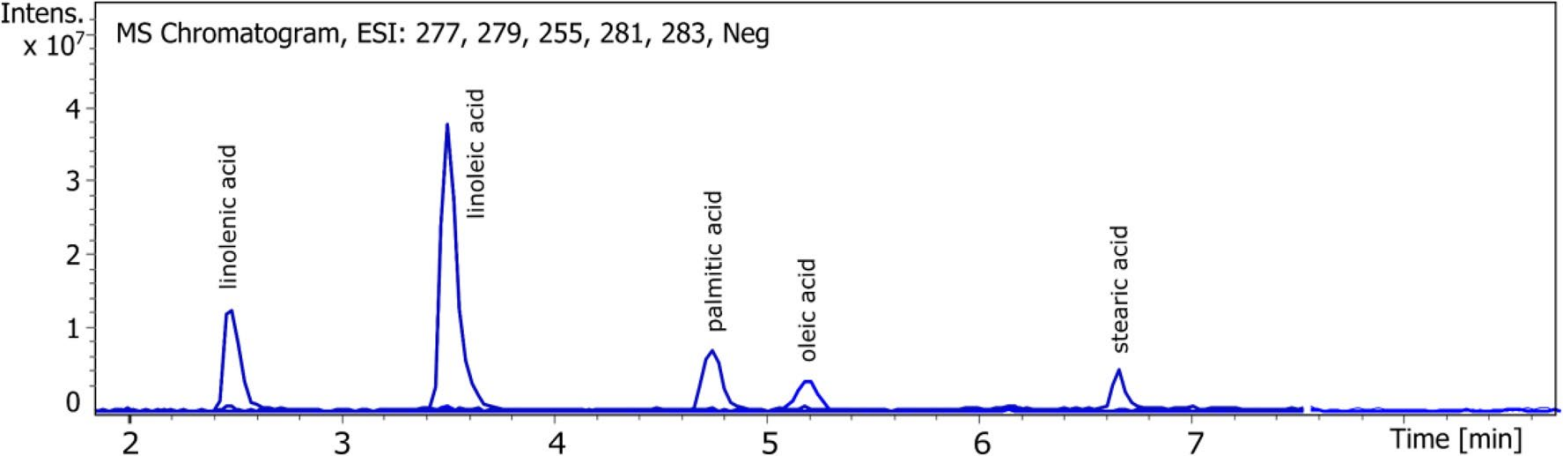
UPLC chromatograph of fatty acids.

**Figure 7:**
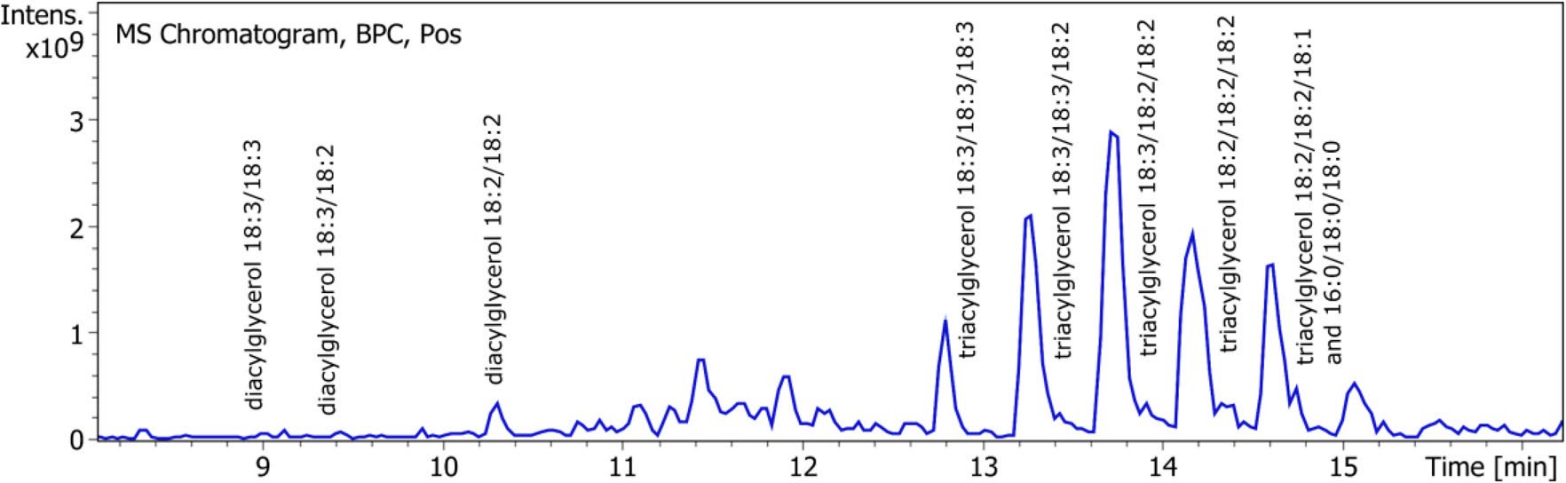
UPLC chromatograph of di and triacylglycerols.

One method able to qualify and quantify free fatty acids, di and triacylglycerols in one time, without any derivatization has been developed with UPLC-DAD-ESI–MS. By using negative mode detection during the first 7 min, free fatty acids are detected: linolenic, linoleic, palmitic, oleic and stearic acids constituting the mean fatty acids in our extract. Parent ([M − H]^−^) ions are detected: m/z 277 for linolenic acid, m/z 279 for linoleic acid, m/z 255 for palmitic acid, m/z 281 for oleic acid and m/z 283 for stearic acid. Only the two polyunsaturated acids give UV signal at 210 nm: linoleic and linolenic acids. After 7 min, detection in positive mode allows to identify di and triacylglycerol by the adduct ion [M + NH_4_]^+^ and the fragments ion showing the loss of fatty acid. In order to know the composition of each di and triacylglycerol, MS2 was used and allowed identification of the neutral losses corresponding to the loss of fatty acid. (Figs. 6, 7 and Table 2).

**Table 2:** Identification of neutral lipids (fatty acids, di and triacylglycerols) found in ASE resin.

Name	Formula	[M − H]^−^, [M + H]^+^, [M + NH_4_] +	MS2 fragments (m/z)	Standard equivalent
Linolenic acid	C_18_H_30_O_2_	277.2 [M − H]^−^		Linolenic acid
Linoleic acid	C_18_H_32_O_2_	279.2 [M − H]^−^		Linoleic acid
Palmitic acid	C_16_H_32_O_2_	255.2 [M − H]^−^		Palmitic acid
Oleic Acid	C_18_H_34_O_2_	281.2 [M − H]^−^		Oleic acid
Stearic acid	C_18_H_36_O_2_	283.3 [M − H]^−^		Stearic acid
Diacylglycerol 18:3/18:3	C_39_H_64_O_5_	630.8 [M + NH_4_]^+^, 613.8 [M + H]^+^	335.8 [M + H–C_18_H_32_O_2_]^+^	Trilinolein
Diacylglycerol 18:3/18:2	C_39_H_66_O_5_	632.8 [M + NH_4_]^+^, 615.8 [M + H]^+^	337.8 [M + H–C_18_H_32_O_2_]^+^, 335.8 [M + H–C_18_H_30_O_2_]^+^	Trilinolein
Diacylglycerol 18:2/18:2	C_39_H_68_O_5_	635.5 [M + NH_4_]^+^, 617.9 [M + H]^+^	337.8 [M + H–C_18_H_32_O_2_]^+^	Trilinolein
Triacylglycerol 18:3/18:3/18:3	C_57_H_92_O_6_	890.8 [M + NH_4_]^+^, 873.7 [M + H]^+^	595.7 [M + H–C_18_H_30_O_2_]^+^	Trilinolein
Triacylglycerol 18:3/18:3/18:2	C_57_H_94_O_6_	892.8 [M + NH_4_]^+^, 875.7 [M + H]^+^	597.7 [M + H–C_18_H_30_O_2_]^+^, 595.7 [M + H–C_18_H_32_O_2_]^+^	Trilinolein
Triacylglycerol 18:3/18:2/18:2	C_57_H_96_O_6_	894.8 [M + NH_4_]^+^, 877.7 [M + H]^+^	599.6 [M + H–C_18_H_30_O_2_]^+^, 597.7 [M + H–C_18_H_32_O_2_]^+^	Trilinolein
Triacylglycerol 18:2/18:2/18:2 (Trilinolein)	C_57_H_98_O_6_	896.5 [M + NH_4_]^+^, 879.7 [M + H]^+^	599.6 [M + H–C_18_H_32_O_2_]^+^	Trilinolein
Triacylglycerol 18:0/18:1/18:1	C_57_H_100_O_6_	898.9 [M + NH_4_]^+^, 881.9 [M + H]^+^	601.7 [M + H–C_18_H_32_O_2_]^+^, 599.7 [M + H–C_18_H_34_O_2_]^+^	Trilinolein
Triacylglycerol 16:0/18:0/18:0	C_55_H_98_O_6_	873.8 [M + NH_4_]^+^, 855.9 [M + H]^+^	599.6 [M + H–C_16_H_32_O_2_]^+^, 575.7 [M + H–C_18_H_32_O_2_]^+^	Trilinolein

Thus m/z 631, 633 and 635 are assigned to diacylglycerol (DAG) 18:3/18:3, DAG 18:3/18:2 and DAG 18:2/18:2.m/z 891, 893, 895 and 897 are assigned to triacylglycerol (TAG) 18:3/18:3/18:3, TAG 18:3/18:3/18:2, TAG 18:3/18:2/18:2 and TAG 18:2/18:2/18:2 (trilinolein). Co-eluated compounds at m/z 899 are assigned to TAG 18:0/18:1/18:1 and TAG 18:2/18:2/18:1 by MS2 fragments.

Neutral lipids comprise between 14.1% and 26.3%; 20.0 ± 5.1% of the ASE resin in average. Free fatty acids represent 34.5% of lipids (6.9 ± 1.8% of the resin) in average (ranging from 4.9 to 9.0% of the resin), diacylglycerols 5.0% (1.0 ± 0.2% of the resin; between 0.8 and 1.2% of the resin) and triacylglycerols 56% (12.2 ± 4.8% of the resin; between 4.3 and 18.6% of the resin). Linoleic, linolenic and palmitic acids are the major free fatty acids; diacylglycerol 18:2/18:2 is the mean diacylglycerol; triacylglycerols 18:3/18:3/18:2, 18:3/18:2/18:2 and 18:2/18:2/18:2 are the major triacylglycerols. For the first time, details of the composition of neutral lipids are given. This work completes Schloman team’s identification of triacylglycerols and free fatty acids^15^: resin was obtained by percolation with acetone (after a first extraction with boiling water), from three and four-year-old 593 (corresponding to CL3 variety here) shrubs. A percentage of 13% of triglycerides in the resin was determined by high-efficiency gel permeation chromatography, with trilinolein as the external standard. Despite the slight difference which may be due to the extraction technique as well as the cultivation conditions, fatty acids found after saponification correspond to those of the triglycerides and free fatty acids: linoleic, linolenic, palmitic, oleic and stearic. Those fatty acids, in particular linolenic and linoleic, are also found in guayule rubber particles^30,31^ under the triacylglycerols form, mainly linear and including mostly unsaturated but also in hydroxy-functional structures. The neutral lipids found in the resin might be derived from the rubber particles.

### Argentatins

A recent paper gives details of 12 cycloartane- and lanostane-type triterpenoids^21^. All these molecules are defined here as belonging to the argentatin family. A simple UPLC-HRMS method was developed to quantify the argentatin family. Low energy ionization was used so the fragments found differ from those given in previous publications.

**Figure 8:**
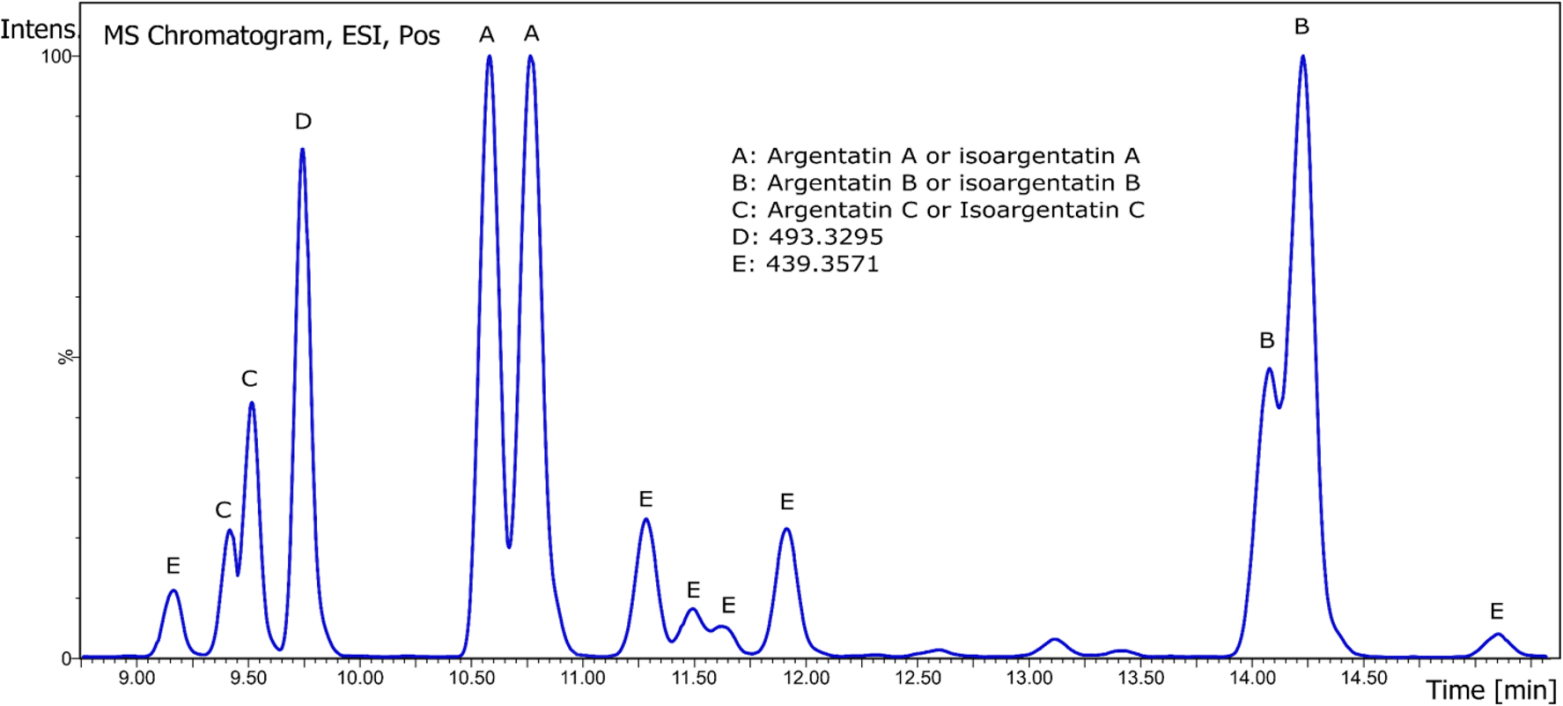
BPI profile of argentatins.

Argentatin A and Isoargentatin A (incanilin) (C_30_H_48_O_4,_), were identified by presence of salt adduct 495.345 [M + Na]^+^, molecular ion 473.364 [M + H]^+^, as well as single, double and triple water loss fragment 455.353 [M − H_2_O]^+^, 437.344 [M − 2H_2_O]^+^, 419.331 [M − 3H_2_0]^+^. Furthermore, a little difference between both spectrawith one containing 313.252 fragment and another 245.191 fragment (group A on Fig. 8, retention times: 9.6 and 9.7 min). The standard used contains a mix of Argentatin A (major) and Isoargentatin A (minor): ^13^C-NMR shifts were the exact same are those given by Komorowski^19^.

Similarly, for Argentatin B and Isoargentatin B (C_30_H_48_O_3_), identification was carried out on the basis sodium adduct presence, molecular ion and water loss fragment: 479.349 [M + Na]^+^, 457.369 [M + H]^+^, 439.357 [M − H_2_O]^+^, 421.347 [M − 2H_2_O]^+^ (group B on Fig. 8, retention time: 11.3 min) A comparison of ^13^C-NMR showed that the standard used was a mix of Argentatin B (major) and Isoargentatin B (minor)^19^. Others compounds sharing the same major fragment (439.357). and with similar profile( 479.349 [M + Na]^+^, 457.369 [M + H]^+^, 439.357 [M − H_2_O]^+^, 421.347 [M − 2H_2_O]^+^) were detected. Some have 229.141 and 279.093 fragments and others 245.191 fragment. They probably correspond to the molecules (C_30_H_48_O_3_) described by Xu^21^: 16-Deoxyargentatin A [(20S,24R)-20,24-Epoxy-25-hydrox-ycycloartan-3-one]; 16-Deoxyisoargentatin A [(20S,24R)-20,24-Epoxy-25-hy-droxylanost-8-en-3-one]; 24-Epi-argentatin H [(16β,20R,24S)-16,24-Dihydroxycy-cloart-25-en-3-one]; Isoargentatin H [(16β,20R,24R)-16,24 Dihydroxylanosta-8,25-dien-3-one]; Argentatin H [(16β,20R,24R)-16,24-Dihydroxycycloart-25-en-3-one] (group E on Fig. 8) but in the absence of pure standards, complete characterisation could not be achieved.

For Argentatin C and Isoargentatin C, similar adduct and fragmentation patterns as Argentatin A and Isoargentatin A were found (497.361 [M + Na]^+^, 457.365 [M − H_2_O]^+^, 437.344 [M − 2H_2_O]^+^, 421.347 [M − 3H_2_0]^+^) and only one contains the 219.176 fragment. (group C on Fig. 8, retention times: 8.9 and 9.1 min).

A peak with *m/z* 493.329 seems to be associated to a molecule similar to argentatins A and C. Adduct, molecular ion and water loss pattern (493.329 [M + Na]^+^, 471.345 [M + H]^+^, 453.336 [M − H_2_O]^+^ and 435.326 [M − 2H_2_O]^+^) fits the description of 16,17(20)-Didehydroargentatin C [(20R,24R)-24,25 Dihy-droxycycloart-17-en-3,16-dione]^21^ (group D on Fig. 8, retention time: 9.3 min). (Table 3).

**Table 3:** Identification of argentatins in ASE resin.

Name	Formula	[M + Na]^+^, [M + H]^+^	Fragments	Standard equivalent
Argentatin A	C_30_H_48_O_4_	495.345 [M + Na]^+^, 473.364 [M + H]^+^	455.353 [M + H–H_2_O]^+^, 437.344 [M + H–2H_2_O]^+^, 419.331 [M + H–3H_2_0]^+^	Argentatin A
Isoargentatin A	C_30_H_48_O_4_	495.345 [M + Na]^+^, 473.364 [M + H]^+^	455.353 [M + H–H_2_O]^+^, 437.344 [M + H–2H_2_O]^+^, 419.331 [M + H–3H_2_0]^+^	Argentatin A
Argentatin B	C_30_H_48_0_3_	479.349 [M + Na]^+^, 457.369 [M + H]^+^	439.357 [M + H–H_2_O]^+^, 421.347 [M + H–2H_2_O]^+^	Argentatin B
Isoargentatin B	C_30_H_48_0_3_	479.349 [M + Na]^+^, 457.369 [M + H]^+^	439.357 [M + H–H_2_O]^+^, 421.347 [M + H–2H_2_O]^+^	Argentatin B
Argentatin C	C_30_H_50_O_4_	497.361 [M + Na]^+^, 457.365 [M + H–H_2_O]^+^	437.344 [M + H–2H_2_O]^+^, 421.347 [M + H–3H_2_0]^+^	Argentatin A
Isoargentatin C	C_30_H_50_O_4_	497.361 [M + Na]^+^, 457.365 [M + H–H_2_O]^+^	437.344 [M + H–2H_2_O]^+^, 421.347 [M + H–3H_2_0]^+^	Argentatin A
Unknown	C_30_H_46_O_3_	493.329 [M + Na]^+^, 471.345 [M + H]^+^,	453.336 [M + H–H_2_O]^+^, 435.326 [M + H–2H_2_O]^+^	Argentatin A

7-Oxoisoargentatin A [(16β,20S,24R)-20,24-Epoxy-16,25-dihydroxylanost-8-en-3,7-dione] (C_30_H_47_O_5_) was not found within the extract.

Argentatin D and Isoargentatin D (C_30_H_50_O_3_, exact mass: 458.3760), quisquagenin, 3-Epi quisquagenin, and Isoquisquagenin (C_30_H_51_O_4_, exact mass: 475.3787), cyclofoetigenin A ((C_30_H_52_O_4_, exact mass: 476.3866), 24-O-p-AnisoylargentatinC[(16β,20R,24R)-24-p-Anisoyl-16,25-dihydroxycycloartan-3-one] (C_38_H_57_O_6_, exact mass: 609.4155) and 24-O-trans-Cinnamoylargentatin C [(16β,20R,24R)-24-trans-Cinnamoyl-16,25-dihydroxycycloartan-3-one] (C_39_H_57_O_5_, exact mass: 605.4206) were not detected in these samples.

**Figure 9:**
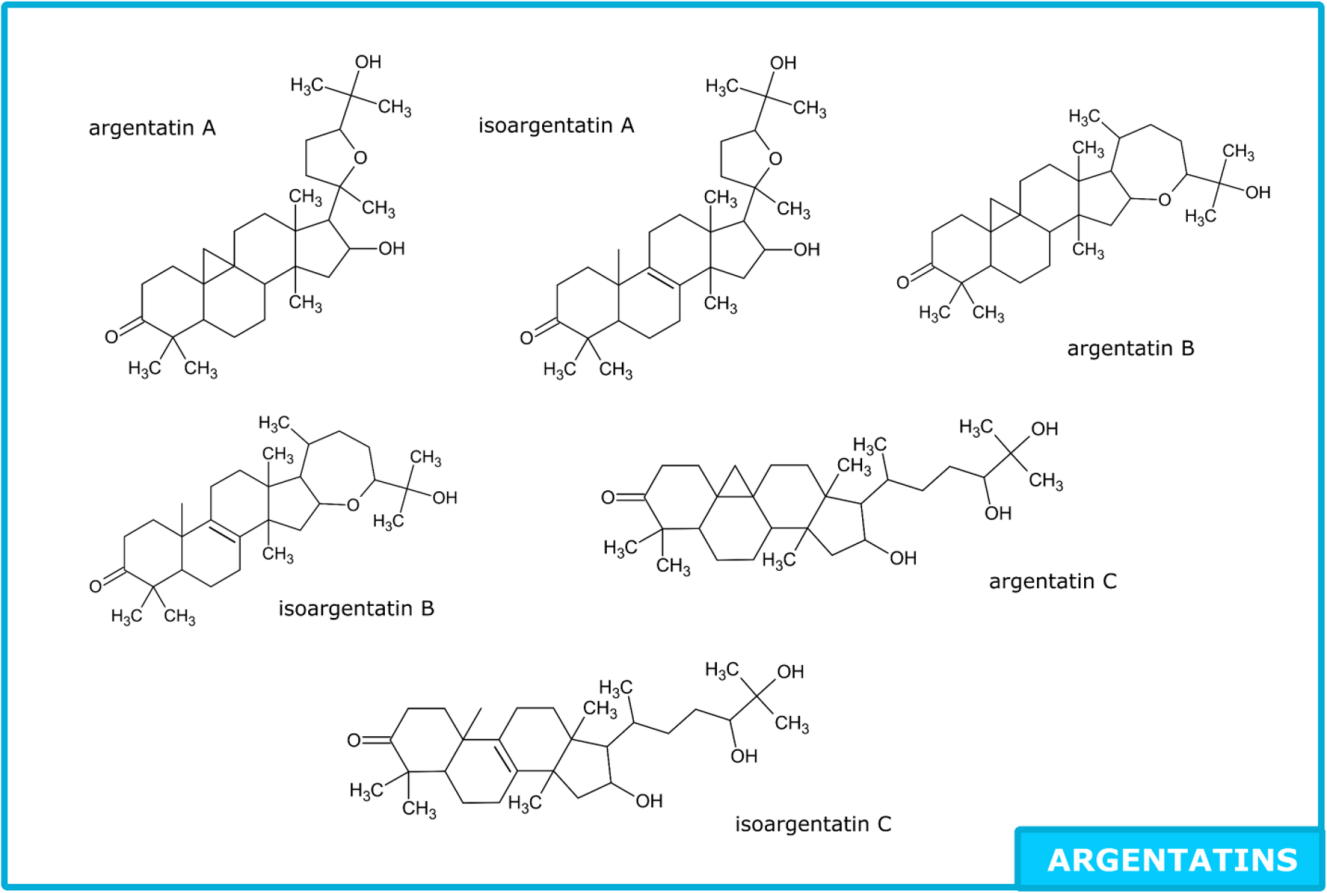
Molecules of argentatin family found in ASE resin.

Resins contain between 40.8% and 67.7% of argentatins/isoargentatins, 56.4 ± 7.6% in average. The major ones are argentatins/isoargentatins A and B, accounting for 29.9 ± 7.2% and 24.3 ± 6.9% in average respectively (Fig. 9) . Schloman^15^ and his team found only 27% of total triterpenoids in a resin extracted with cold acetone (after previous boiling water extraction) from three and four-year-old 593 guayule plants (corresponding to CL3 variety here). This difference can be due to the extraction method. Indeed, Komoroski^19^ writes that it is usually about 55% of argentins that is found in guayule resin from southern Texas and northern Mexico.

### Alkanes and alkanals

**Figure 10:**
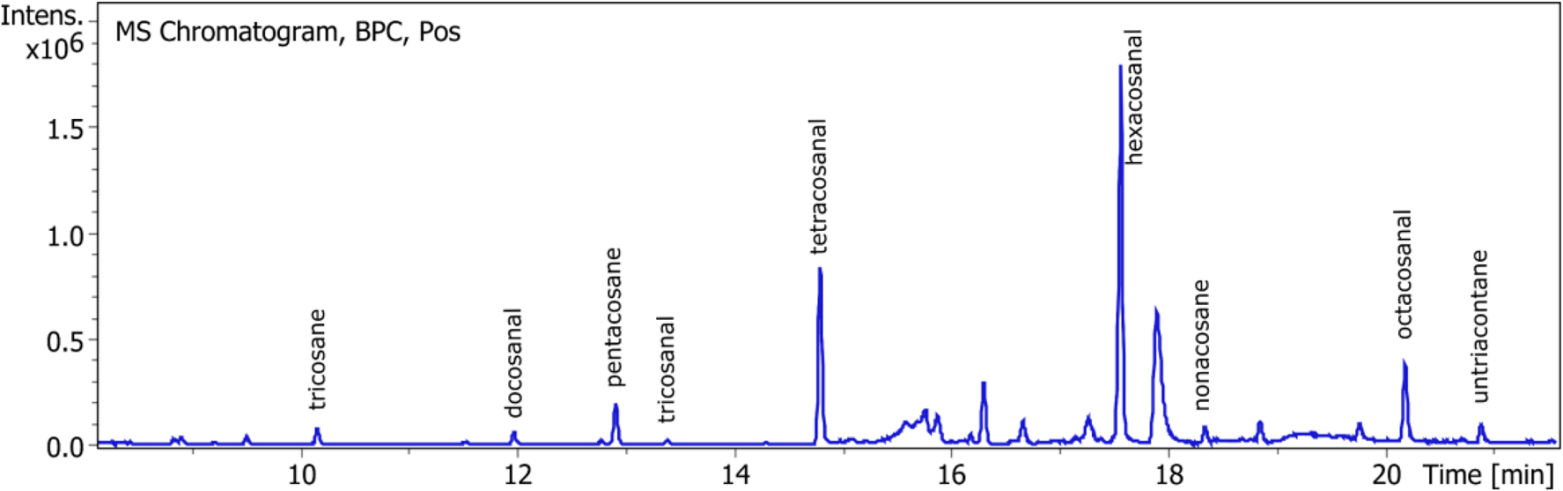
GC chromatograph of alkanes and alkanals.

Waxes were analyzed by gas chromatography in order to determine their composition. Alkanes and alkanals were recognizable with their spectra patterns: 57, 71, 85, 99 (alkanes), and 82, 96 and pseudomolecular ion (M − H_2_O) (alkanals). To quantify them, an alkane standard mixture and a dososanal standard were used. (Fig. 10 and Table 4).

**Table 4:** Identification of alkanes and alkanals in ASE resin.

Name	Formula	[M]	Fragments	Standard equivalent
Tricosane	C_23_H_48_	324 [M]	113, 99, 85, 71, 57	Docosane
Pentacosane	C_25_H_52_	352 [M]	113, 99, 85, 71, 57	Tetracosane
Nonacosane	C_29_H_60_	408 [M]	113, 99, 85, 71, 57	Octacosane
Untriacontane	C_31_H_64_	436 [M]	113, 99, 85, 71, 57	Triacontane
Docosanal	C_22_H_44_O		306 [M − H_2_O], 110, 96, 82, 69, 57	Docosanal
Tricosanal	C_23_H_46_O		320 [M − H_2_O], 110, 96, 82, 69, 57	Docosanal
Tetracosanal	C_24_H_48_O		334 [M − H_2_O], 110, 96, 82, 69, 57	Docosanal
Hexacosanal	C_26_H_52_O		362 [M − H_2_O], 110, 96, 82, 69, 57	Docosanal
Octacosanal	C_28_H_56_O		390 [M − H_2_O], 110, 96, 82, 69, 57	Docosanal

Between 2.3% and 8.3% of alkanals were found, 4.9 ± 1.4% in average. The major ones were tetracosanal and hexacosanal. Alkanes were present as traces (> 0.01%). Banigan^23^ described 3–4% of “waxlike substance” in the leaves so our results seems to match with previous analysis. However Marwah^7^ fractionated guayule wax and obtained 3% of alkanes ranging from C19 to C36, 92% of esters and a high polar part.

### Sugars, glycerol and organic acids

**Figure 11:**
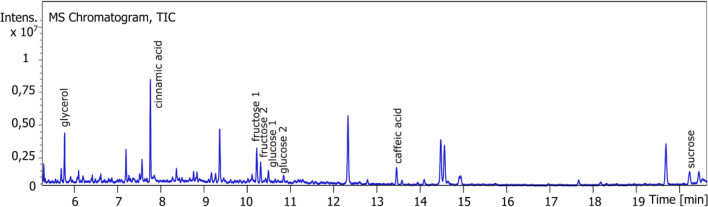
GC chromatograph of sugars, organic acids and glycerol.

Glucose and fructose have already been found in the bagasse^5^ but not in the resin. Glycerol and organic acids have already been described in the resin. Sucrose was added to the identification list and sugars, organic acids and glycerol were quantified. ASE resin was derivatized in two steps: methoxamine hydrochloride (aldehyde and cetone function) and BSTFA (alcool and acid function): MS spectra were identified thanks to NIST database. (Fig. 11 and Table 5).

**Table 5:** Identification of sugars, organic acids and glycerol in ASE resin.

Name	Formula	Fragments	Standard equivalent
Glycerol (3TMS derivative)	C_12_H_32_O_3_Si_3_	299, 218, 205, 147, 117, 103, 73	Glycerol (3TMS derivative)
Cinnamic acid (TMS derivative)	C_12_H_16_O_2_Si	220, 205, 161, 145, 131, 103, 73	Cinnamic acid (TMS derivative)
Fructose (5MS derivative)	C_21_H_52_O_6_Si_5_	364, 307, 277, 217, 204, 147, 133, 103, 73	Fructose (5MS derivative)
Glucose (5MS derivative)	C_21_H_52_O_6_Si_5_	319, 217, 205, 160, 147, 129, 103, 73	Glucose (5MS derivative)
Caffeic acid (3TMS derivative)	C_18_H_32_O_4_Si_3_	396, 381, 219, 191, 103, 73	Caffeic acid (3TMS derivative)
Sucrose (8TMS derivative)	C_36_H_86_O_11_Si_8_	437, 361, 271, 217, 169, 147, 103, 73	Sucrose (8TMS derivative)

The percentage of each sugar is less than 1% but the average sum over all samples is 0.9 ± 0.4% (between 0.3 and 1.5%). The major ones are fructose and sucrose. Caffeic acid content is really low (> 0.2%). Cinnamic acid accounts for 4.1 ± 1.5% in average (between 1.6 and 6.5%). Glycerol content is 1.0 ± 0.8% (between 0.3 and 3.3%).

### Monoterpenes and phytosterols

As the plants were dried at 50 °C and stored at room temperature for a few days, the analysis of monoterpenes is not performed in this study. The volatile part of the ASE resin (mainly α and β-pinene) usually accounts for between 2 and 4%^25^. Analysis of monoterpenes and phytosterols (β-sitosterol and stigmasterol are present in guayule^15,32^) can be carried out using GC.

## Conclusions

This article provides chromatographic methods for the characterization of guayule resin, a complex mixture of organic chemicals produced by acetone extraction of the guayule plant. The work is of interest to researchers working with the crop, to those working in natural product characterization methods, and for general interest in plant secondary metabolites.

To the author’s knowledge, it is the first time that the majority of compounds are analyzed on the same resin. Analytical methods used are principally MS methods, customized to allow detection of multiple metabolites simultaneously. Guayule resin contains hundreds to thousands of chemicals and with this work, supplemental informations are given (new polyphenols, identification of alkanes, alkanals, sugars, di and triacylglycerols) (Table 6).

**Table 6:** Average percentages of molecule found in ASE resin (in 4-year-old plants, CL1 and CL3 varieties) and extremums between brackets (biological variety).

Family of molecule	Molecule or group of molecules	Average and extremums percentages found in ASE resins
Polyphenols	3–O–caffeoylquinic, 4–O–caffeoylquinic 5–O–caffeoylquinic, 3,4–O–dicaffeoylquinic, 3,5–O–dicaffeoylquinic and 4,5–O–dicaffeoylquinic acids	1% [0.01–0.9%]
Guayulins	Guayulins (A, B, C, D)	8.6% [4.1–12.1%]
	Guayulin A	3.6% [0.9–5.4%]
	Guayulin B	0.4% [0.05–1.9%]
	Guayulin C	4.1% [1.6–6.6%]
	Guayulin D	0.5% [0.3–0.8%]
Neutral lipids	Free fatty acids, di and triacylglycerols	20.0% [14.1–26.3%]
	Free fatty acids	6.9% [4.9–9.0%]
	Diacylglycerols	1.0% [0.8–1.2%]
	Triacylglycerols	12.2% [4.3–18.6%]
Argentatines	Argentatins/isoargentatins and derivatives	56.4% [40.8–67.7%]
	Argentatin A and Isoargentatin A	29.9% [14.4–54.7%]
	Argentatin B and Isoargentatin B	24.3% [4.3–38.1%]
Alkanals	Docosanal, Tricosanal, Tetracosanal, Hexacosanal, Octacosanal	4.9% [2.3–8.3%]
Sugars	Fructose, glucose, sucrose	0.9% [0.3–1.5%]
	Cinnamic acid	4.1% [1.6–6.5%]
	Glycerol	1.0% [0.3–3.3%]

**Figure 12:**
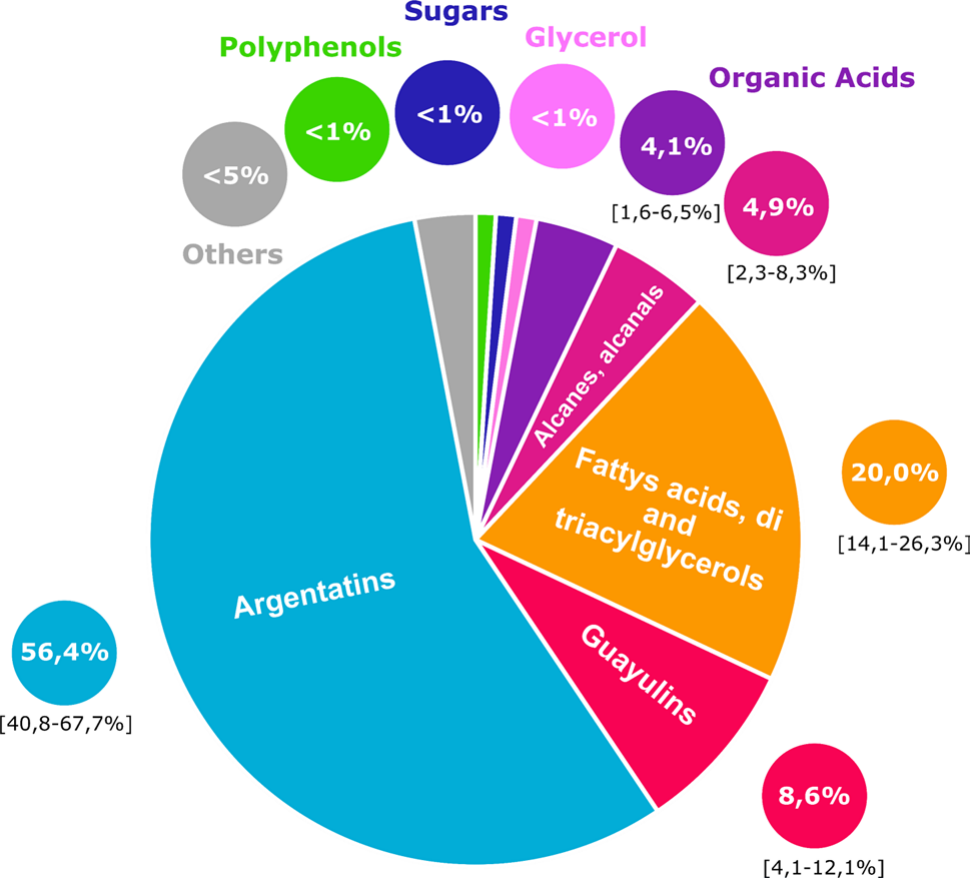
Average percentages of molecule families in guayule ASE resin (in 4-year-old plants, CL1 and CL3 varieties) and extremums between brackets (biological variety).

Samples of various varieties and differing plant age have been analyzed in order to ensure not to miss any important compounds. To give an idea of possible contents, average percentages are given for each family of molecules (Fig. 12). These percentages can help other scientists in their experiments on guayule and given methods allows them to analyze their own resin.

The stated objective (identifying and quantifying all components of the same resin) is mostly achieved: less than 5% of compounds remain unqualified and quantified. Knowing the composition of guayule resin and the quantity of each molecule is of great interest as many of them have a high added value: guayulins have shown repellent and anti-feedant activities^33^ and argentatins could be used in cancer treatments^6^. The aim is now to extract and fractionate this resin to valorise it.

## Data avilabilty

The datasets analyzed during the current study available from the corresponding author on reasonable request.
