# Author Correction: Composition of Guayule (*Parthenium argentatum* Gray) resin

**DOI:** 10.1038/s41598-023-33332-7

**Published:** 2023-04-18

**Authors:** Amandine Rousset, Christian Ginies, Olivier Chevallier, Mariano Martinez-Vazquez, Ali Amor, Michel Dorget, Farid Chemat, Sandrine Perino

**Affiliations:** 1 GuaTecs, 28 Rue Xavier Bichat, 72000 Le Mans, France; 2 Avignon University, INRAE, UMR408, GREEN Extraction Team, 84000 Avignon, France; 3 Avignon University, INRAE, UMR408, MicroNut Team, 84000 Avignon, France; 4 Avignon University, DARI, Plateforme 3A, 84000 Avignon, France; 5 Instituto de Química, Universidad Nacional Autónoma de México, 04510 CDMX, México

Correction to: *Scientific Reports* https://doi.org/10.1038/s41598-023-29524-w, published online 28 February 2023

The original version of this Article contained a repeated error where in several instances the abbreviation of Ultra Performance Liquid Chromatography, ‘UPLC’, was incorrectly stated as ‘ULPC’.

As a result, in the Materials and methods, under the subheading ‘Chromatographic methods’,

“*Argentatins* were determined using a ULPC-MS system Waters with a C18 CSH Waters (100 m × 2.1 mm × 1.7 µm) column.”

now reads:

*“Argentatins* were determined using a UPLC-MS system Waters with a C18 CSH Waters (100 m × 2.1 mm × 1.7 µm) column.”

In addition, in the Results and discussion, under the subheading ‘Free fatty acids, di and triacylglycerols’,

“One method able to qualify and quantify free fatty acids, di and triacylglycerols in one time, without any derivatization has been developed with ULPC-DAD-ESI–MS.”

now reads:

“One method able to qualify and quantify free fatty acids, di and triacylglycerols in one time, without any derivatization has been developed with UPLC-DAD-ESI–MS.”

Furthermore, in the caption of Figure 3,

“ULPC chromatograph of polyphenols and guayulins.”

now reads:

“UPLC chromatograph of polyphenols and guayulins.”

In the caption of Figure 6,

“ULPC chromatograph of fatty acids.”

now reads:

“UPLC chromatograph of fatty acids.”

Lastly, in the caption of Figure 7,

“ULPC chromatograph of di and triacylglycerols.”

now reads:

“UPLC chromatograph of di and triacylglycerols.”

The original Article has been corrected.

